# Supplementary material for: An amplicon-based sequencing framework for accurately measuring intrahost virus diversity using PrimalSeq and iVar
Source: Genome Biol. 2019 Jan 8;20:8. doi: 10.1186/s13059-018-1618-7 (PMC6325816; doi:10.1186/s13059-018-1618-7)
Supplement: Supplementary file 2 — iSNV frequencies calculated using pseudo replicates (Figure S1.), sensitivity of measuring intrahost diversity at 5% (Figure S2.), metagenomic sequencing coverage depth (Figure S3.), and validation of iVar for intrahost single-nucleotide variant calling (Figure S4.), consensus calling (Figure S5.), and trimming (Figure S6.). (PDF 7109 kb) [file 13059_2018_1618_MOESM2_ESM.pdf]

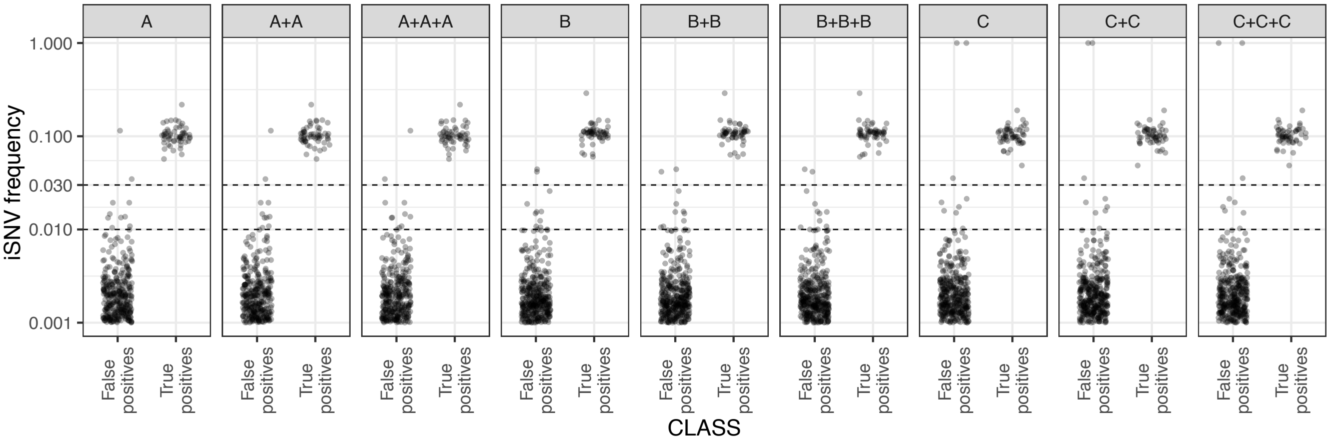

Figure S1. iSNV frequencies calculated using pseudo replicates. iSNVs filtered using the same replicate instead of technical replicates. The "ivar filtervariants" command was used to filter variants by supplying the iSNVs found in the same replicate multiple times.

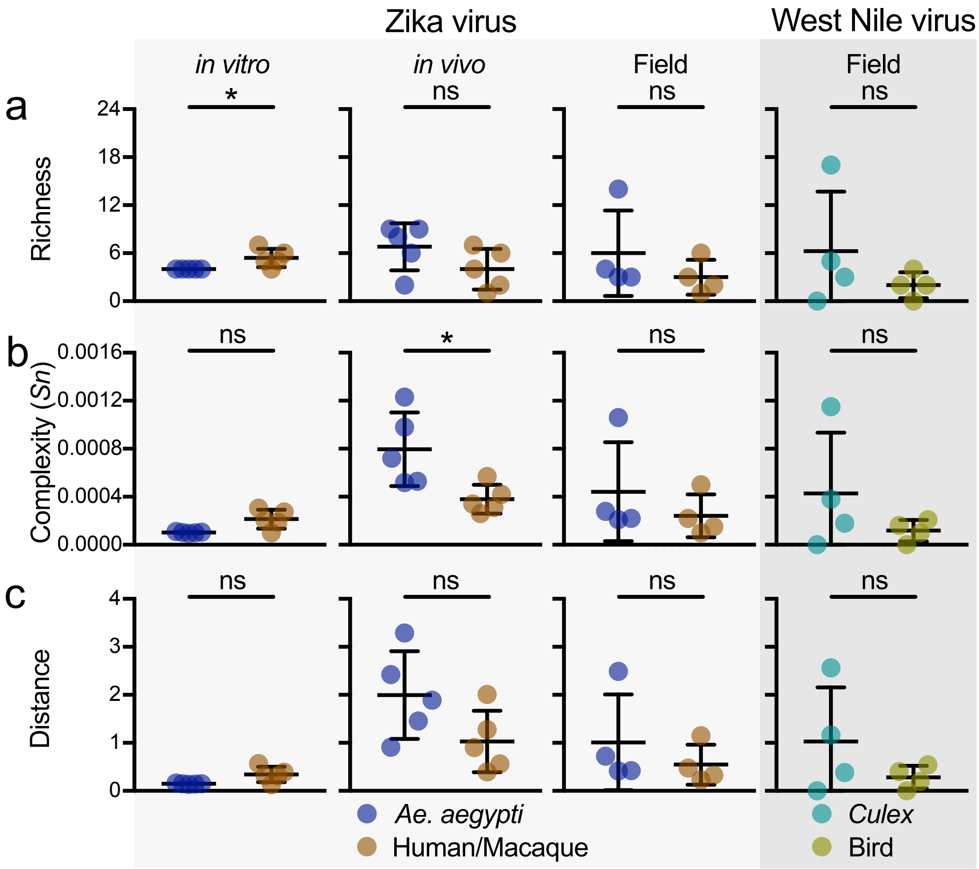

Figure S2. Sensitivity of measuring intrahost virus genetic diversity at a 5% frequency cutoff. Variants >5% called from Zika and West Nile virus populations derived from *in vitro*, *in vivo*, and field studies were used to compare intrahost virus diversity and selection from mosquito vectors (*Ae. aegypti* and *Culex* species) and vertebrate hosts (primates or birds). We compared (**a**) richness (the number of intrahost single nucleotide variant [iSNV] sites; Fig. 7a), (**b**) complexity (uncertainty associated with randomly sampling an allele, measured by Shannon entropy [*Sn*]), and (**c**) distance (the sum of all iSNV frequencies). The mosquito and vertebrate-derived populations were compared using unpaired Mann-Whitney rank t-tests (ns, not significant; *, $p < 0.05$). Data shown as mean and standard deviation.

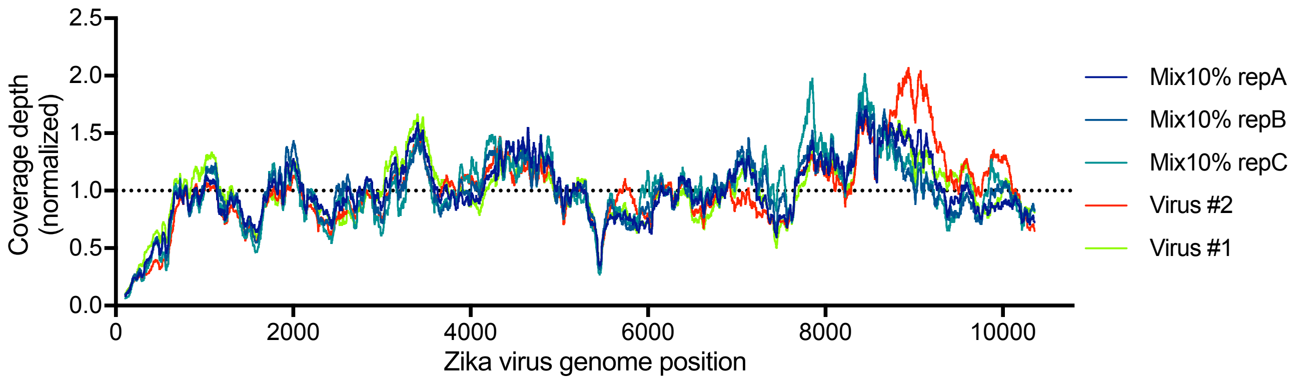

Figure S3. Normalized coverage depth of the Zika virus genome using metagenomics sequencing. Normalized coverage depth was calculated by the depth per position divided by the mean depth across the coding sequence.

Validation of iVar for intrahost single nucleotide variant calling, consensus calling, and trimming.

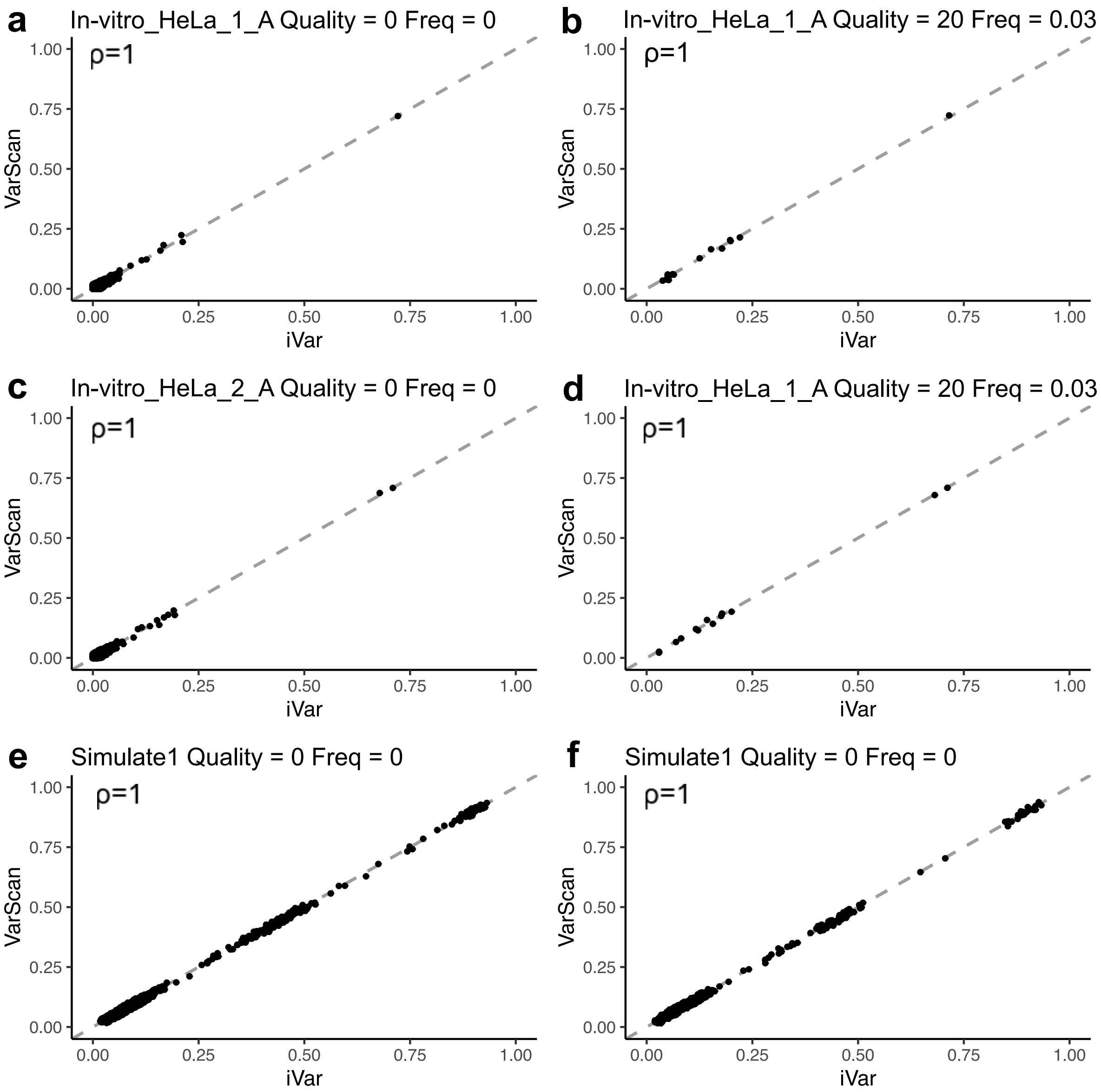

Figure S4. Validation of intrahost single nucleotide variant calling in iVar. Variant calling using iVar was validated against the `mpileup2snp` and `mpileup2indel` commands in VarScan 2.3.9 using four datasets. "Field_Human_1_A" and "Field_Human_2_A" are clinical Zika virus samples collected during the 2016 outbreak in Florida and sequenced using the PrimalSeq protocol. "Simulate 1" and "Simulate2" are 150 nt pair ended read simulated using wgsim (github.com/lh3/wgsim) at an error rate of 10% and a mutation rate of 1% and 2%, respectively. Because VarScan assumes a diploid genome and shows only the intrahost single nucleotide variant (iSNV) allele with maximum frequency at a given position, the iSNV called by iVar were filtered to choose the one with maximum frequency per position and the frequencies were plotted against each other. (**a**) iSNVs called on Field_Human_1_A with quality threshold 0 and iSNV frequency threshold of 0. (**b**) iSNVs called on Field_Human_1_A with quality threshold 20 and iSNV frequency threshold of 0.03. (**c**) iSNVs called on Field_Human_2_A with quality threshold 0 and iSNV frequency threshold of 0. (**d**) iSNVs called on Field_Human_2_A with quality threshold 20 and iSNV frequency threshold of 0.03. (**e**) iSNVs called on Simulate1 with quality threshold 0 and iSNV frequency threshold of 0. (**f**) iSNVs called on Simulate2 with quality threshold 0 and iSNV frequency threshold of 0.

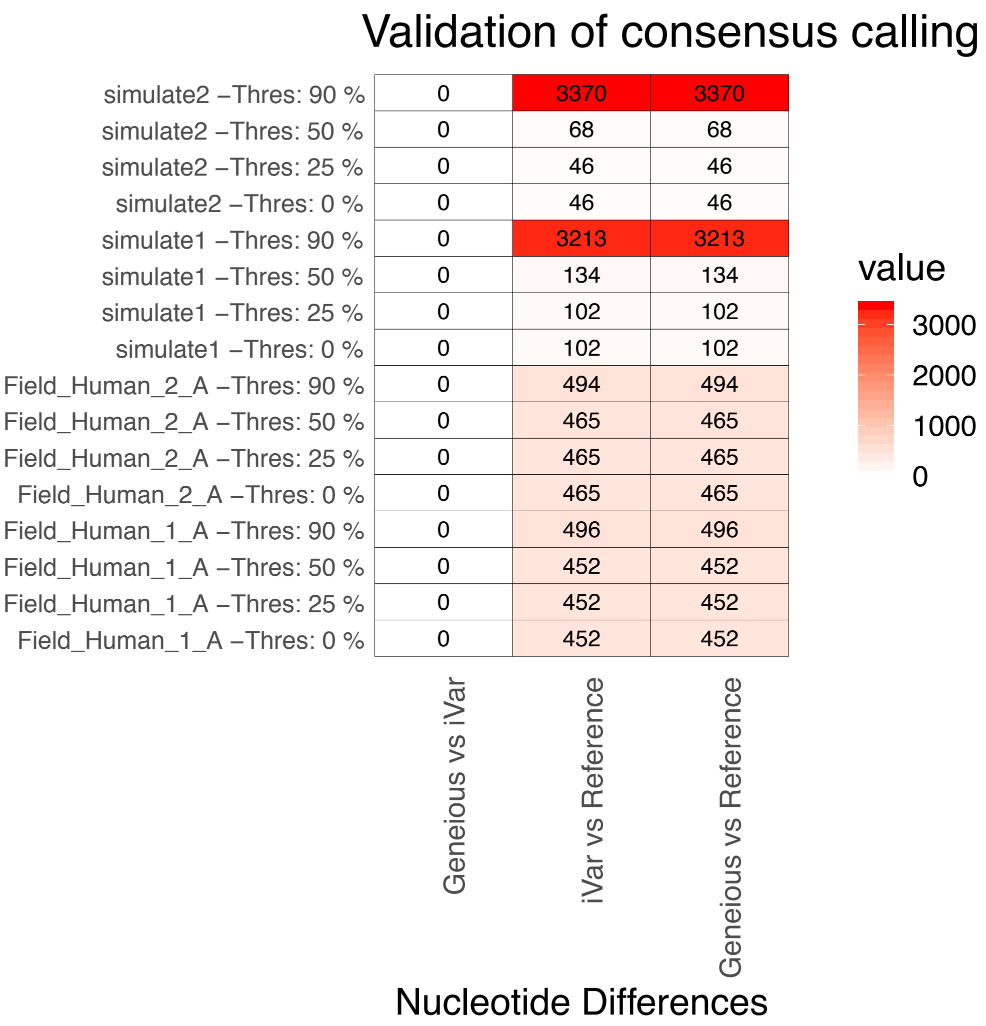

Figure S5. Validation of consensus calling in iVar. Consensus genome calling using iVar was validated against the consensus calling using Geneious using four datasets at four thresholds - 0%, 25%, 50% and 90%. "Field_Human_1_A" and "Field_Human_2_A" are clinical Zika virus samples collected during the 2016 outbreak in Florida and sequenced using the PrimalSeq protocol. "Simulate 1" and "Simulate2" are 150 nt pair ended read simulated using wgsim (github.com/lh3/wgsim) at an error rate of 10% and a mutation rate of 1% and 2%, respectively. The resulting consensus sequences from iVar, Geneious, and the reference sequence, used to generate the alignment, were then aligned to each other. The differences in nucleotide positions (mismatches + gaps) in the resulting multiple sequence alignment are shown in the figure. "Thres." = "Threshold".

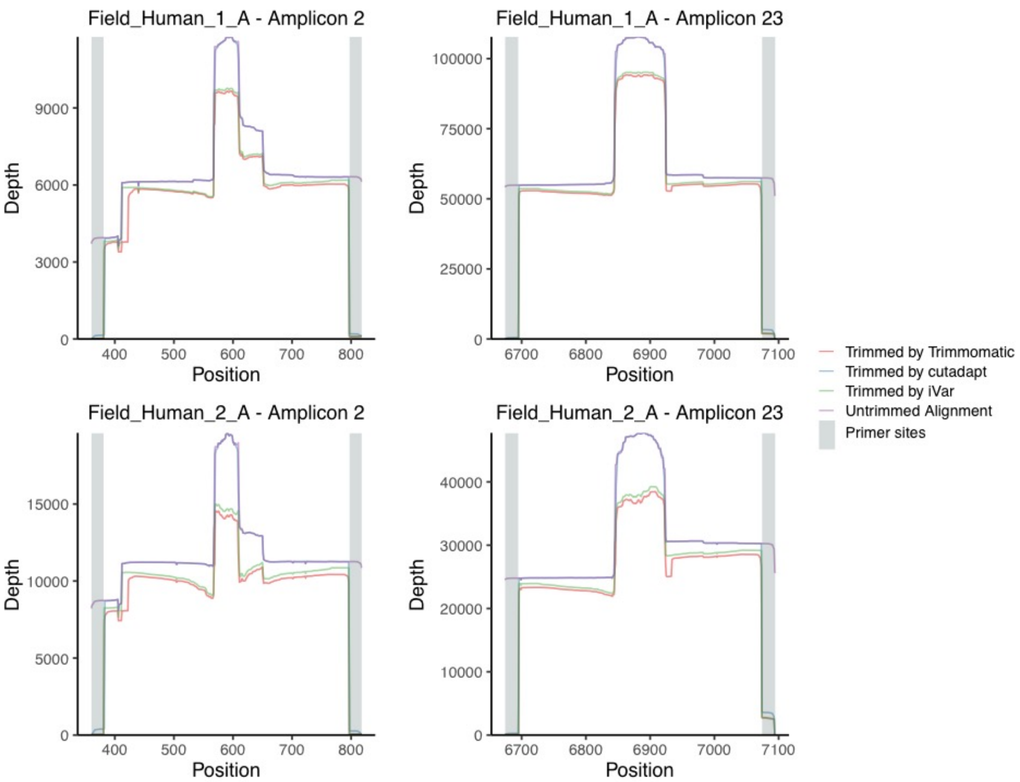

Figure S6. Validation of primer and quality trimming in iVar. Primer and quality trimming in iVar was validated against anchored adapter trimming using cutadapt. "Field_Human_1_A" and "Field_Human_2_A" are clinical Zika virus samples collected during the 2016 outbreak in Florida and sequenced using the PrimalSeq protocol. Reads from both samples that aligned to amplicons numbers 2 and 23 (**see Fig. 2**) were extracted. To generate the depth per position curve for cutadapt, reads were trimmed using cutadapt by supplying the primer sequences and aligned to the reference genome. To generate the depth per position curve for iVar, the reads were aligned to the reference genome and then trimmed using iVar by supplying primer positions in a bed file. To generate the depth per position curve for Trimmomatic, the first 22 bp from the 5'end of every read were trimmed using the 'HEADCROP' option. The threshold for quality trimming in all three cases was set to 20 and minimum read length threshold was set to 30. The grey bands highlight the primer regions.
