# Supplementary material for: An amplicon-based sequencing framework for accurately measuring intrahost virus diversity using PrimalSeq and iVar
Source: Genome Biol. 2019 Jan 8;20:8. doi: 10.1186/s13059-018-1618-7 (PMC6325816; doi:10.1186/s13059-018-1618-7)
Supplement: Supplementary file 3 — Laboratory protocol for generating sequencing libraries for measuring intrahost virus genetic diversity. (PDF 198 kb) [file 13059_2018_1618_MOESM3_ESM.pdf]

An amplicon-based sequencing framework for accurately measuring intrahost virus diversity using PrimalSeq and iVar

Nathan D Grubaugh, Karthik Gangavarapu, Joshua Quick, Nathaniel L. Matteson, Jaqueline Goes De Jesus, Bradley J Main, Amanda L Tan, Lauren M Paul, Doug E Brackney, Saran Grewal, Nikos Gurfield, Koen KA Van Rompay, Sharon Isern, Scott F Michael, Lark L Coffey, Nicholas J Loman, Kristian G Andersen

For updates, see grubaughlab.com/open-science/amplicon-sequencing/
Contact information: Nathan Grubaugh (nathan.grubaugh@yale.edu)

The general approach to this protocol is to amplify the virus genome in small (~400 bp) overlapping fragments using two highly multiplexed PCR reactions (where the overlapping segments are in separate reactions). The amplicons are combined after PCR and are the correct size for library preparation and paired-end 250 nt sequencing using the Illumina MiSeq.

Primers are listed in Additional file 2 and grubaughlab.com/open-science/amplicon-sequencing/. Or build your own using Primal Scheme: primal.zibraproject.org/

Notes:

1. Requires at least 1000 virus RNA copies going into cDNA synthesis. More is better. Try to normalize virus RNA copies between samples to make comparisons easier.
2. Process each RNA sample twice through the protocol to sequence as technical replicates. By calling iSNVs only present in both replicates, it reduces the number of false positives (mainly from sequencing errors) and increases the accuracy of iSNV frequency measurements.
3. Obtain at least 400x nt coverage of each nucleotide position. Because of different amplicon efficiencies, this typically means that ~1M 250 nt paired-end reads are needed. Amplification of high input virus concentrations (>10,000 virus RNA copies) are more even and require fewer total reads.
4. During our validation process, the lowest iSNV frequency that we could accurately and consistently measure was 3%. Measuring lower than this requires additional input copies, coverage depth, and validation.
5. Beware of iSNVs that exist within primer binding sites as they can decrease the amplification efficiency of that particular virus haplotype. Because the primer sites are trimmed and are covered by an overlapping amplicon, the iSNVs within the primer sites can be accurately measured. All iSNVs within the amplicon with a primer mismatch, however, can be significantly altered. This is the major limitation with any PCR protocol for virus population diversity analysis.
6. Use our data pipeline, iVar (intrahost variant analysis from replicates) to process and analyze the data. It will align to the reference (or call a consensus), trim primers, call variants, compare variants between replicates, and flag variants within primer sites.

Overview of the PrimalSeq and iVar protocols

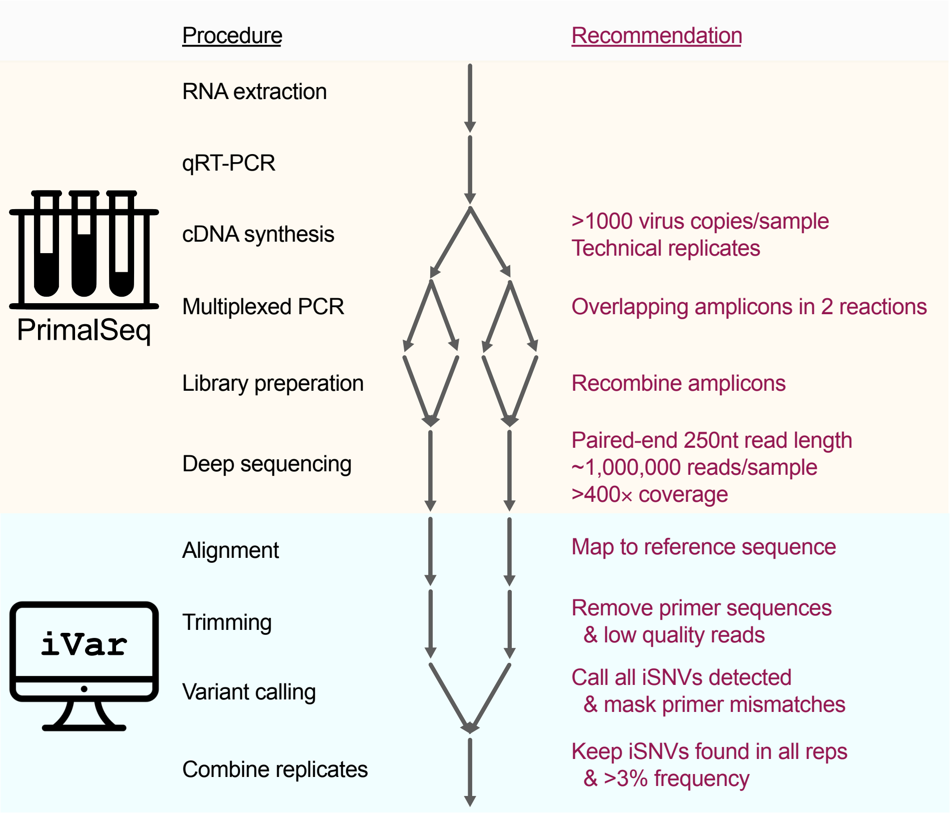

Preparation of cDNA (est. time: 1 hour)
Reagents:
    SuperScript IV VILO Master Mix

Generation of tiled amplicons (est. time: 5 hours)
Reagents:
    Q5 High-Fidelity 2X Master Mix
    Custom primers
    Qubit High Sensitivity dsDNA kit

Library preparation and quantification (est. time: 3-4 hours)
Reagents:
    KAPA HyperPrep kit (¼ recommended reagents)
    Mag-Bind TotalPure NGS
    BIOO Scientific NEXTflex Dual-Indexed DNA Barcodes
    Qubit High Sensitivity dsDNA kit
    High Sensitivity DNA Analysis Kit
    KAPA Library Quantification kit (optional)
    MiSeq Reagent kit v2 (500 cycle output) or v3 (600 cycle output)

Data analysis
Software:
    iVar: github.com/andersen-lab/ivar

Notes: *Alternate reagents can be used at several steps – please contact us if you have questions. Add no-template water controls at each of the cDNA and PCR steps to monitor for contamination.*

Preparation of cDNA

1. Isolate viral RNA using Omega Viral DNA/RNA kit, Trizol, or equivalent.
2. Many different cDNA synthesis kits can be used, but chose something that is relatively high-fidelity. The current protocols uses SuperScript IV VILO Master Mix because the enzyme has low error rates and the protocol is fast and easy.

**Component**	**Volume in 20 µL reaction**
SSIV VILO Master Mix	4 µL
Nuclease-free water	6-15 µL
Virus RNA	1-10 µL

3. Run the following cycles on a thermocycler:

**Temperature**	**Time**
25°C	10 minutes
50°C	10 minutes
85°C	5 minutes
4°C	∞

4. Store samples at 4°C (same day) or -20°C (up to a week) until ready for PCR.

PCR generation of tiled amplicons

1. Validated primer schemes can be found: grubaughlab.com/open-science/amplicon-sequencing/. Prepare two primer pools by mixing equal volumes of each 10 μM primer, or follow the specific notes for each primer set.
2. Prepare two PCR reactions for each sample (one for each primer pool):

**Component**	**Volume in 25 μL reaction**
Q5 2x Master Mix	12.5 μL
Primer pool (#1 or #2)	1 μL
Nuclease-free water	9.5 μL
cDNA	2 μL

3. Run the following cycles on a thermocycler:

**Temperature**	**Time**
98°C	30 seconds
95°C	15 seconds
65°C	5 minutes
Repeat steps 2 & 3 for a total of **35 cycles**	
4°C	∞

4. Run 5 μL of each product on a 1% agarose gel. Each should produce a visible 400 bp band.

Post PCR cleanup (1.8:1 ratio of beads to sample) and quantification

1. Allow Mag-Bind TotalPure NGS beads to equilibrate to room temperature, vortex until homogenous.
2. Bring PCR product volume up to 25 μL with water (if not at volume already).
3. Add 45 μL of beads to 25 μL of PCR product, mix well, and incubate at room temperature for 10 minutes.
4. Place tubes on a magnetic stand and incubate until solution appears clear.
5. Discard supernatant without disturbing the beads.
6. While tubes are on the magnet, add 200 μL of 80% EtOH, incubate for 30 seconds, and discard the EtOH wash.
7. Repeat previous 80% EtOH wash and remove as much EtOH as possible.
8. Leave tubes on magnet and air dry for 5 minutes.
9. Remove tubes from magnet and add 20 μL of nuclease-free water. Mix well by pipetting.
10. Place tubes on magnet stand. When solution appears clear, remove supernatant without disturbing the beads and place into new tubes.
11. Quantify the DNA concentration using the Qubit High Sensitivity DNA kit (or equivalent) from 1 μL of each product. Expected range = 10-100 ng/μL DNA. Sequencing from lower concentrations may still work.
12. *Note: If your lab has a KingFisher, automated protocols are available upon request.*

Library preparation (using ¼ of vendor recommended reagents)

End-repair

1. Combine 25-50 ng of PCR-amplified DNA from primer pool 1 and 2 together for a total of 50-100 ng in 12.5 µL (equal concentrations of each amplicon pool). QS to a total volume of 12.5 µL using nuclease-free water.
   Alternatively: proceed using 50-100 ng of primer pool product separately for library preparation. This allows for additional monitoring of cross-contamination. Data can be merged computationally post sequencing.
2. Combine the following components from the Kapa Hyper prep kit for end repair:

**Component (¼ reagents)**	**Volume in 15 µL reaction**
End Repair & A-tailing buffer	1.75 µL
End Repair & A-tailing enzyme mix	0.75 µL
PCR-amplified DNA (50 ng)	12.5 µL

3. Run the following cycles on a thermocycler:

**Temperature**	**Time**
20°C	30 minutes
65°C	30 minutes
4°C	∞

Adaptor ligation

1. Dilute a working stock of NEXTflex Dual-Indexed DNA Barcodes 1:100 to obtain a concentration of 250 nM. Select unique barcodes for each sample. Try not to repeat barcodes from recent runs.
   Note: Be careful to not cross-contaminate the adaptors by centrifuging all liquid from the caps and only opening one index at a time.
2. Combine the following components:

**Component (¼ reagents)**	**Volume in 27.5 µL reaction**
Ligation buffer	7.5 µL
DNA ligase	2.5 µL
NEXTflex DNA Barcodes (250nM)	2.5 µL
End repair reaction product	15 µL

3. Incubate at 20°C for 15 minutes.
4. Proceed immediately to cleanup.

Post ligation cleanup (0.8:1 ratio of beads to sample)

1. Allow Mag-Bind TotalPure NGS beads to equilibrate to room temperature, vortex until homogenous.
2. Add 22 µL of beads to 27.5 µL of ligation product, mix well, and incubate at room temperature for 10 minutes.
3. Place tubes on a magnetic stand and incubate until solution appears clear.
4. Discard supernatant without disturbing the beads.
5. While tubes are on the magnet, add 200 µL of 80% EtOH, incubate for 30 seconds, and discard the EtOH wash.
6. Repeat previous 80% EtOH wash and remove as much EtOH as possible.
7. Leave tubes on magnet and air dry for 5 minutes.
8. Remove tubes from magnet and add 20 µL of nuclease-free water. Mix well by pipetting.
9. Place tubes on magnet stand. When solution appears clear, remove supernatant without disturbing the beads and place into new tubes - 15 µL will go into library amplification.
10. *Note: If your lab has a KingFisher, automated protocols are available upon request.*

Library amplification

1. Combine the following components:

**Component**	**Volume in 34 µL reaction**
2X KAPA HiFi HotStart ReadyMix	17 µL
Illumina primer mix	2 µL
Adaptor-ligated library	15 µL

2. Run the following cycles on a thermocycler:

**Temperature**	**Time**
98°C	45 seconds
98°C	15 seconds
60°C	30 seconds
72°C	30 seconds
Repeat steps 2-4 for a total of **8 cycles**	
72°C	1 minute
4°C	∞

3. Proceed directly to cleanup or store at 4°C.

Post amplification cleanup (0.8:1 ratio of beads to sample)

1. Allow Mag-Bind TotalPure NGS beads to equilibrate to room temperature, vortex until homogenous.
2. Add 32 µL of beads to 40 µL of amplified product, mix well, and incubate at RT for 10 minutes.
3. Place tubes on a magnetic stand and incubate until solution appears clear.
4. Discard supernatant without disturbing the beads.
5. While tubes are on the magnet, add 200 µL of 80% EtOH, incubate for 30 seconds, and discard the EtOH wash.
6. Repeat previous 80% EtOH wash and remove as much EtOH as possible.
7. Leave tubes on magnet and air dry for 5 minutes.
8. Remove tubes from magnet and add 25 µL of 10 mM **Tris-EDTA (TE)** buffer. Mix well by pipetting.
9. Place tubes on magnet stand. When solution appears clear, remove supernatant without disturbing the beads and place into new tubes.
10. *Note: If your lab has a KingFisher, automated protocols are available upon request.*

Sequencing preparation

Library quantification and pooling

1. Quantify the DNA concentration of each sample (1 µL) using the Qubit High Sensitivity DNA kit.
2. Pool equal concentrations (*e.g.*, 1-10 ng) of each library for sequencing.
3. Check DNA fragment distributions of the pooled sample using the BioAnalyzer DNA 1000 kit. Peak fragment size from 400 bp tiled amplicons with proper ligated adaptors should be ~ 580 nt. If ~180 bp bands (adaptor dimers) still exist, perform post amplification cleanup again.
4. Quantify the DNA concentration of the pooled library (1 µL) using the Qubit High Sensitivity DNA kit.
5. *Note: At least 0.76 ng/µL is required to achieve 2 nM for library pooling. Libraries will need to be concentrated or re-amplified if less than this amount.*
6. Convert DNA libraries from weight to moles:
   Molecular weight [nM] = Library concentration [ng/µL] / ((ave. library size x 650)/1,000,000)
   Example: if ave. size of library is 580 bp and concentration is 2.5 ng/µL…
   (580 x 650) / 1,000,000 = 0.377
   2.5 / 0.377 = 6.6 nM
7. Dilute the pooled library to 2 nM in 10 mM TE.
8. (Optional) Ensure the library molar concentration using the Kapa Library Quantification kit.
9. **If sending your sample to a genomics core (*i.e.*, not loading the MiSeq yourself), stop here.**

Diluting the pooled library for sequencing

1. Combine 10 µL of the 2nM pooled library to 10 µL of 0.1 N NaOH and mix. Incubate from 5 minutes at room temperature to denature the dsDNA.
2. Add 980 µL of HT1 (comes with the MiSeq kits). New concentration = 20 pM.
3. Dilute to the desired concentration using the following volumes.

**Concentration**	**10 pM**	**12 pM**	**14 pM**	**16 pM**
20 pM Library	295 µL	355 µL	415 µL	475 µL
Prechilled HT1	300 µL	240 µL	180 µL	120 µL
PhiX control*	5 µL	5 µL	5 µL	5 µL

*PhiX control should also be denatured and diluted to 20 pM.

4. *Note: loading too high of a sample on a MiSeq leads to over-clustering and decreased quality, which may make the data unusable. Adding too low leads to under-clustering and may not generate enough data for sufficient sequencing coverage. In our hands, optimal cluster densities were reached using 10-12 pM with the MiSeq v2 kits and 14-16 pM with the MiSeq v3 kits. Loading concentrations should be empirically determined with each lab.*
5. Following loading instructions located in the MiSeq user guides.

Data processing and analysis

1. Use iVar, follow the instructions on: github.com/andersen-lab/ivar
